# Using qualitative methods, what factors do child & adolescent higher trainees and new consultants (within 1 year of appointment), report in choosing their higher training subspecialty?

**DOI:** 10.1192/bjo.2021.746

**Published:** 2021-06-18

**Authors:** Mary Parker, Jim Boylan, Nicholas Wolstenholme

**Affiliations:** Tees, Esk & Wear Valley NHS Foundation Trust

## Abstract

**Aims:**

This Research aims to identify factors reported by recent and current trainees in choosing Child & Adolescent (C&A) Psychiatry for their higher training/career path. The hypothesis is that via thematic analysis prominent factors will emerge to inform future recruitment and retention.

**Background:**

The need to attract more doctors into Psychiatry has been identified by NHS, Royal College of Psychiatrists and the Media; Health Education England figures show core psychiatry had lowest fill rates of any specialty in 2016,17 &18.

Some subspecialties experience particular difficulties e.g. C&A. Royal College of Psychiatry analysis of workforce in March 2018 showed the numbers of C&A psychiatrists of all grades have fallen by 6.3% in four years and for consultants alone the decrease was 6.9% over the same period.

However, very little research has been completed investigating why trainees might choose C&A Psychiatry with a literature search revealing only one report of C&A trainees views in the UK in 2006.

**Method:**

A qualitative design was chosen to provide insight into the factors affecting participants in choosing their career.

The theoretical framework supporting the study relates to capturing experience via a case study approach, aiming to explore reported issues in a real life context and considering similarities in the cases to inform future recruitment.

The Research was approved by The Health Research Authority and local Research & Development departments.

Purposeful sampling was used with voluntary participation following informed consent and non-identifiable demographic data were collected and analysed quantatively.

Semi-structured interviews to saturation were conducted with fourteen (N = 14) participants asked questions exploring their subspecialty choice. Responses were recorded and transcribed verbatim prior to thematic analysis, including triangulation via a co-coding process to check areas were not over-represented and/or subject to researcher thoughts and possible unconscious biases.

**Result:**

Semi-structured interviews included all North-East C&A trainees and 3 recently qualified Consultants.

Demographics results included 5 male and 6 female participants and 6 non-UK graduates.

Emergent themes were analysed into key findings including:

- interest in children/specialty
- specialty experience
- supportive supervisors/team
- work-life balance
- opportunity to impact/intervene early.

Limitations include study of a single geographical area and possible researcher bias.

**Conclusion:**

This Research provides novel findings re factors influencing career choice of C&A Psychiatry to inform future recruitment and retention. Clear themes have emerged re important recruitment/retention factors and the study highlights need for more research to investigate reasons why C&A is not chosen.

